# The synergic effects of frailty on disability associated with urbanization, multimorbidity, and mental health: implications for public health and medical care

**DOI:** 10.1038/s41598-018-32537-5

**Published:** 2018-09-20

**Authors:** Wei-Ju Lee, Li-Ning Peng, Chi-Hung Lin, Hui-Ping Lin, Ching-Hui Loh, Liang-Kung Chen

**Affiliations:** 1 Aging and Health Research Center, National Yang Ming University, Taipei City, Taiwan; 2 Department of Geriatric Medicine, School of Medicine, National Yang Ming University, Taipei City, Taiwan; 3 Department of Family Medicine, Taipei Veterans General Hospital Yuanshan Branch, Yilan County, Taiwan; 4 Center for Geriatrics and Gerontology, Taipei Veterans General Hospital, Taipei City, Taiwan; 5 Department of Health, New Taipei City Government, New Taipei City, Taiwan; 6 Center of Health and Aging, Hualien Tzu Chi Hospital Buddhist Tzu Chi Medical Foundation, Hualien County, Taiwan

## Abstract

Frailty is garnering increasing interest as a potential target in disability prevention. Since it is uncertain how frailty interacts with multimorbidity, urbanization, and mental health to affect disability, we investigated the epidemiology of frailty and its synergies with these factors. The study enrolled 20,898 participants aged 65 and older living in New Taipei city. All participants received face to face interview to assess frailty, multimorbidity, urban or rural residence, and mental health. Individual versus combined effects of risk factors were evaluated using the Rothman synergy index. Prevalence of frailty was 5.2% overall, 7.2% in multimorbid participants, 9.6% in rural residents, and 20.8% in those with mental disorders. Logistic regression, adjusted for age and sex, showed significant associations between disability and frailty (OR 8.5, 95% CI 6.4–11.2), multimorbidity (OR 1.3, 95% CI 1.0–1.6), urbanization (OR 1.3, 95% CI 1.0–1.7), and mental disorders (OR 7.3, 95% CI 5.6–9.5); these factors had a significant synergic effect on disability. Frailty is common in older adults and associated with disability, and was synergetic with multimorbidity, mental disorders, and residing rurally. Targeting frailty prevention and intervention needs a special attention on those vulnerable groups.

## Introduction

With increasing life-expectancy and decreasing fertility, population aging has become a global phenomenon and is occurring more rapidly in Asia than elsewhere. This demographic transition has profound impacts on health care, financial, and social systems. The World Health Organization has proposed a framework for healthy aging, and encourages policymakers and health professionals to preserve and maximize old people’s intrinsic capacities, as well as to destigmatize dependence in older age^1^. For centuries, a single-disease perspective of nosology and pathophysiology has prevailed in medicine. However, it is difficult to apply a simple disease-centred model to address contemporary challenges posed by the diversity and complexity of old people and population aging; rather, comprehensive public health strategies that integrate functional preservation and disease management are needed^2^. To respond better to emerging needs in an ageing society with complex care requirements, researchers and clinicians must shift from a single disease mindset toward a more multimorbidity-focused approach^3^. In this way, the health care system can evolve from an index disease model of care towards a more person-centric model, in which patients receive simultaneous management for multiple comorbidities^4^.

Frailty is a geriatric syndrome distinct from multimorbidity, characterized by increasing vulnerability and decreasing physical reserve due to accumulating multisystem deficits^5^. Moreover, frailty is a dynamic state, often considered as a predisposing factor in disability, and therefore of public health interest as a potential target for disability prevention^6^. Although the phenotypic definition of frailty proposed by Fried *et al*.^5^ focuses primarily on physical condition, others contend that mental health status modifies the impact of frailty on disability and mortality^7,8^; a similar synergistic impact of mental health disorders in multimorbidity has also been reported^3^.

There are disparities in health and mortality between people of different socioeconomic status and who live in rural rather than urban areas^9–12^. It is uncertain, however, whether or not frailty is more prevalent in rural areas or interacts synergistically with low urbanization to affect functional dependence, and what potential implications there may be in terms of the prevalence and rate of population aging. Understanding such differences may help policymakers and health professionals to mobilize resources and develop customized strategies for preventing and managing disability. Hence, this study evaluated the impact of frailty on disability and investigated whether urbanization and mental health may exert synergistic effects.

## Results

### Study participants

**Figure 1:**
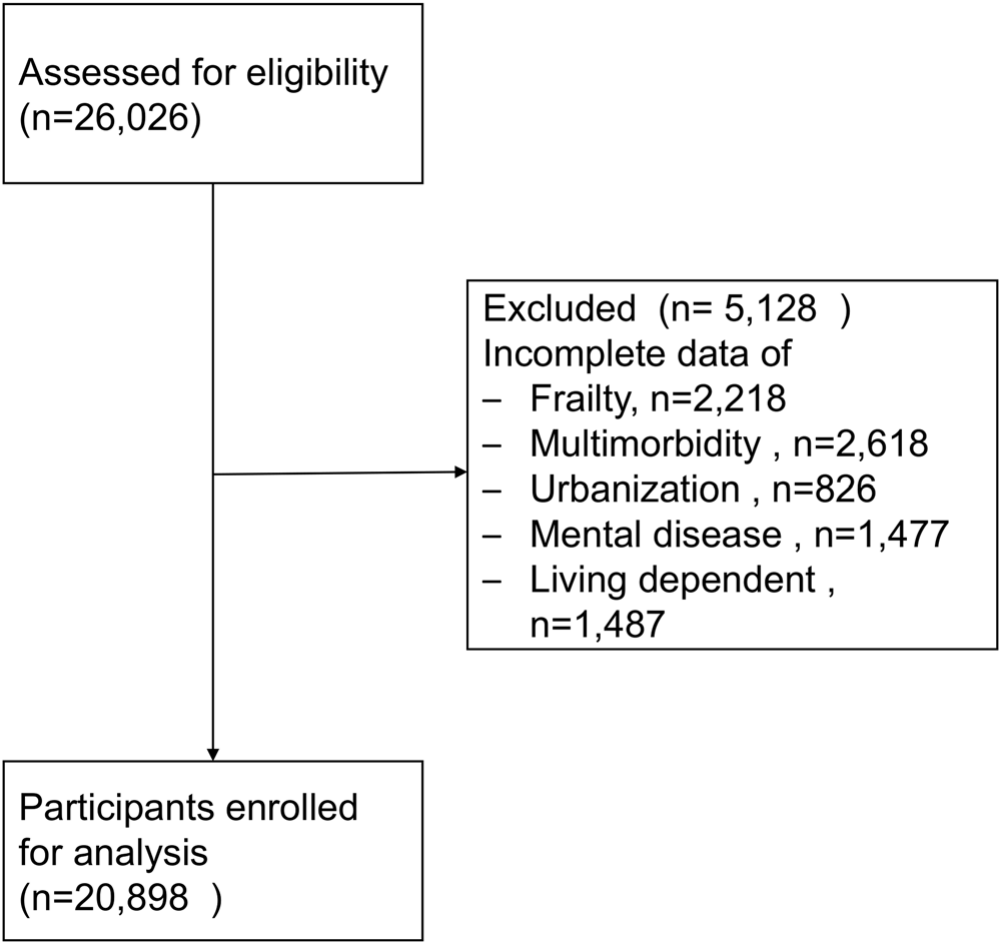
consort flowchart for participants’ enrollment in the study.

After eligibility screening, 20898 Elderly Health Examination Database registrants were enrolled (Fig. 1); compared to this analytic cohort, 5128 excluded due to incomplete data were older (74.0 *vs* 72.6 years, p < 0.001), more often female (59.3% *vs* 52.9%, p < 0.001), and included relatively lower proportions who smoked (2.5 *vs* 6.2%, p < 0.001) or consumed alcoholic drinks (9.8% *vs* 11.0, p = 0.012).

**Figure 2:**
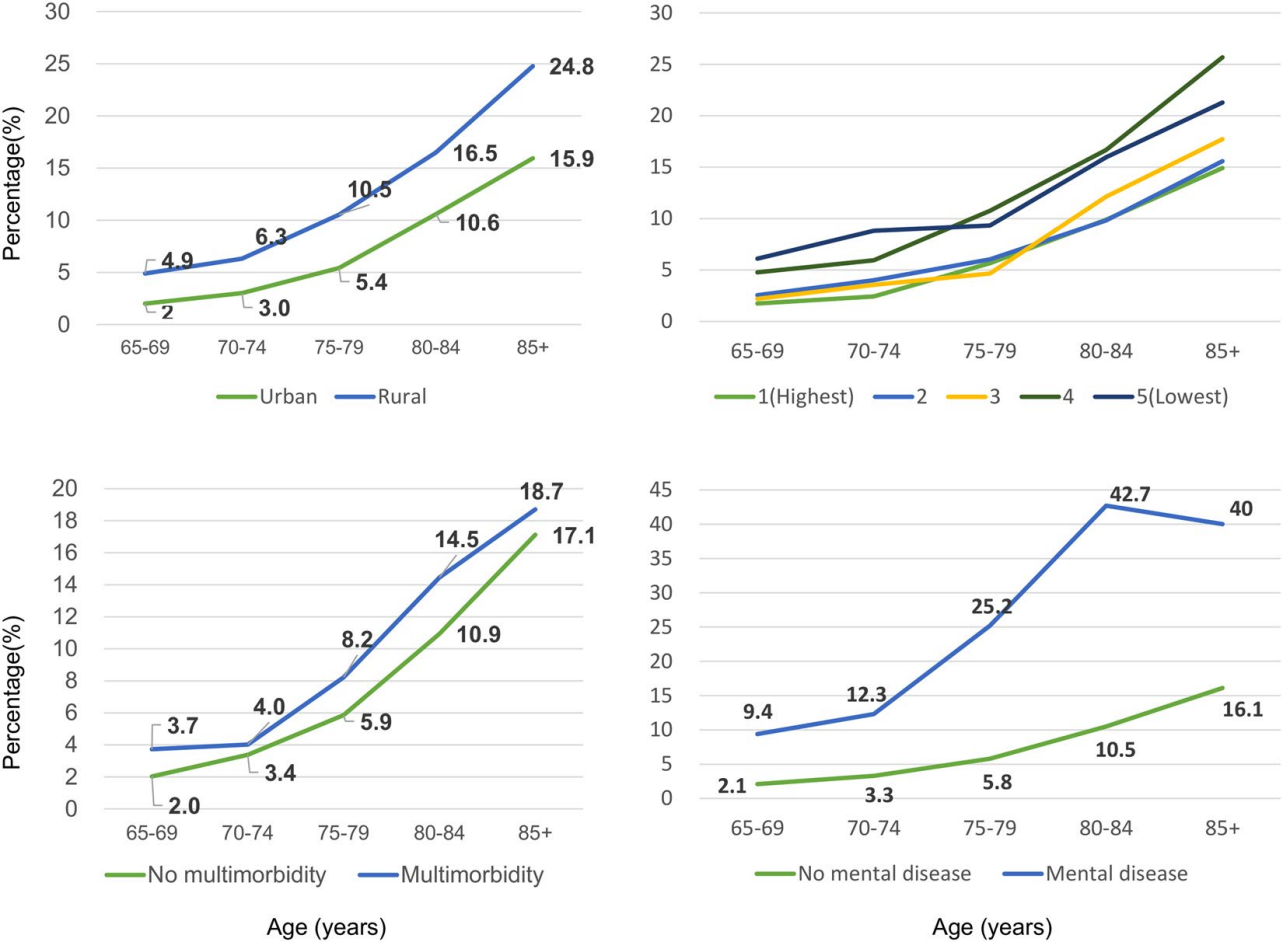
Prevalence of frailty in different age groups stratified by urbanization, multimorbidity and mental disease.

The majority of participants were more than 70 years old, and women (Table 1). Frailty was more prevalent among older 5-year age groups than younger ones. In general, frailty was more prevalent among vulnerable subjects, i.e. those with multimorbidity (7.2%) or mental disorders (20.8%), and residing rurally (9.7%). Incident frailty was advanced by 5 years in less urbanized areas, and mental disorders by 10 years (Fig. 2).

**Table 1:** Demographic characteristics, frailty, multimorbidity, and mental disease.

Data values show number (%)	Total frequency	Frailty status				Multimorbidity			Mental disorder		
		Robust	Prefrail	Frail	*p*-value	No	Yes	*p*-value	No	Yes	*p*-value
Age (years)					<0.001			<0.001			<0.001
65–69	8558(41.0)	4926(47.3)	3434(36.5)	198(18.3)		7136(42.8)	1422 (33.6)		8344(41.2)	214(33.0)	
70–74	5321(25.5)	2892(27.8)	2242(23.8)	187(17.3)		4201(25.2)	1120 (26.5)		5166(25.5)	155(23.9)	
75–79	3821(18.3)	1704(16.4)	1873 (19.9)	244(22.6)		2969(17.8)	852 (20.1)		3698(18.3)	123(19.0)	
≥80	3198(15.3)	888(8.5)	1859 (19.8)	451(41.8)		2358(14.2)	840 (19.8)		3041(15.0)	157(24.2)	
Male	9844(47.1)	5126(49.2)	4267(45.4)	451(41.8)	<0.001	7846(47.1)	1998 (47.2)		9548(47.2)	296(45.6)	0.438
Smoke	1296(6.2)	625(6.0)	602(6.4)	69(6.4)	0.498	1032(6.2)	264(6.2)	0.919	1259(6.2)	37(5.7)	0.591
Taking alcohol	2294(11.0)	1244(12.0)	965(10.3)	85(7.9)	<0.001	1820(10.9)	474(11.2)	0.614	2203(10.9)	91(14.0)	0.012
Multimorbidity	4234(20.3)	2045(19.6)	1885(20.0)	304(28.2)	<0.001				4060(20.1)	174(26.8)	<0.001
Rural residence^a^	3055(14.6)	1082(10.4)	1678(17.8)	295(27.3)	<0.001	2294(13.8)	761(18.0)	<0.001	2961(14.6)	94(14.5)	0.921
Disability^b^	419(2.0)	121(1.2)	173(1.8)	125(11.6)	<0.001	310(1.9)	109(2.6)	0.003	340(1.7)	79(12.2)	<0.001
Mental disorder	649(3.1)	183(1.8)	331(3.5)	135(12.5)	<0.001	475(2.9)	174(4.1)	<0.001			

^a^Urbanization level 4 or 5.

^b^Functionally dependent.

### Associations and synergies

Logistic regression adjusted for age and sex, showed significant associations of functional dependence with frailty, multimorbidity, less urban residence, and mental disorders. In multivariate analysis, frailty and mental disorders independently predicted disability (Table 2); however, associations of multimorbidity and urbanization, with disability were not statistically significant.

**Table 2:** Odds ratios for functional dependence associated with corresponding risk factors.

	Frequency	Crude model	Model 1^a^	Model 2^b^
	n (%)	OR (95%CI)	OR (95%CI)	OR (95%CI)
Age (years)				
65–69	124 (29.6)	ref	ref	ref
70–74	71 (17.0)	0.9 (0.7–1.2)	0.9 (0.6–1.1)	0.8 (0.6–1.1)
75–79	81 (19.3)	1.5 (1.1–2.0)	1.2 (0.9–1.6)	1.2 (0.9–1.5)
≥80	143 (34.1)	3.2 (2.5–4.1)	1.9 (1.5–2.5)	1.8 (1.4–2.4)
Male (*vs* female)	192 (45.8)	0.9 (0.8–1.2)	1.0 (0.8–1.2)	1.0 (0.8–1.2)
Age and sex adjusted				
Smoke (*vs* not)	21 (5.0)	0.9 (0.6–1.4)	0.8 (0.5–1.3)	0.9 (0.5–1.4)
Take alcohol (*vs* not)	41 (9.8)	1.0 (0.7–1.4)	1.1 (0.7–1.5)	1.0 (0.7–1.5)
Frailty status				
Robust	121 (28.9)	ref	ref	ref
Prefrail	173 (41.3)	1.4 (1.1–1.8)	1.4 (1.1–1.8)	1.3 (1.1–1.7)
Frail	125 (29.8)	8.5 (6.4–11.2)	8.4 (6.3–11.1)	6.4 (4.8–8.6)
Multimorbidity (*vs* none)	109 (26.0)	1.3 (1.0–1.6)	1.2 (0.9–1.5)	1.2 (0.9–1.5)
Rural residence (*vs* urban)^c^	84 (20.1)	1.3 (1.0–1.7)	1.1 (0.8–1.4)	1.2 (0.9–1.5)
Mental disorder (*vs* none)	79 (18.9)	7.3 (5.6–9.5)		5.0 (3.8–6.6)

OR = odds ratio. CI = confidence interval.

^a^Adjusted for age, sex, smoke, drink, frailty, multimorbidity, and urbanization.

^b^Adjusted for Model 1 plus mental disease.

^c^Rural = urbanization level 4 or 5. Urban = urbanization level 1–3.

Frailty, urbanization, multimorbidity, and mental disease all had significant synergistic effects on disability (Table 3). In the fully adjusted model, those with frailty and mental disorders were nearly 30 times more likely to be disabled, with RERI of 18.9. Mental disorder, multimorbidity and less urbanized residence had a significant synergistic effect on disability.

**Table 3:** Synergistic effects of frailty with urbanization, mental disorders, and multimorbidity on functional dependence.

Adjusted analytic models	Subgroup comparison	Odds Ratio (95% CI)	Relative excess risk due to interaction (95% CI)	Attributable proportion due to interaction (95% CI)	Synergy Index (95% CI)
Model 1:	Robust	ref	1.4 (0.9–2.5)	0.2 (0.2–0.3)	1.3 (1.3–1.4)
	Urban^a^ & frail	5.1 (3.9–6.8)			
	Rural^b^ & non-frail	1.1 (0.8–1.5)			
	Rural^b^ & frail	6.7 (4.6–9.9)			
Model 2	Robust	ref	18.9 (12.6–28.5)	0.7 (0.7–0.7)	3.3 (3.3–3.3)
	No mental disorder & frail	5.3 (4.0–6.9)			
	Mental disorder & non-frail	5.0 (3.4–7.2)			
	Mental disorder & frail	28.2 (19.0–41.6)			
Model 3	Robust	ref	3.6 (1.5–8.0)	0.4 (0.3–0.5)	1.9 (1.6–2.4)
	Non-multimorbidity & frail	4.3 (3.2–5.8)			
	Multimorbidity & non-frail	0.9 (0.7–1.3)			
	Multimorbidity & frail	8.0 (5.6–11.4)			

CI = Confidence interval. Model 1 adjusted for age, sex, smoking, taking alcohol, multimorbidity and mental disorder. Model 2 adjusted for age, sex, smoking, taking alcohol, multimorbidity and urbanization. Model 3 adjusted for age, sex, smoking, taking alcohol, urbanization and mental disorder.

^a^Urbanization level 1–3.

^b^Urbanization level 4 or 5.

The risk associated with less urbanized residence and disability became insignificant upon factoring prevalence of older people in each municipal district into the full model. Less urbanized residence and increasing population age ≥65 years were both significantly associated with disability in the full model.

## Discussion

New Taipei City is the most populous metropolis in Taiwan, with substantial differences between its constituent municipal districts. In a large population-based sample of senior citizens, frailty was common and grew more prevalent with age. This association is unequivocally established, but other findings are new or less well known. First, people living in less urbanized areas, or who had multimorbidity or mental disorders were more vulnerable to physical frailty. Second, besides significant individual associations of disability with frailty, urbanization, multimorbidity, and mental disorders, we detected synergistic effects between frailty and urbanization, and multimorbidity and mental diseases disorders; the synergy between frailty and mental disease had a particularly strong effect on disability risk.

The age-related rise in frailty, and female predominance observed in this study are consistent with previous reports^5,13,14^. At 5.8%, the prevalence of frailty in New Taipei City appears similar to that in other studies that used the phenotypic definition, and much lower than estimated by the accumulated-deficit index method^15^ Slightly lower prevalence than the 6.8% reported by the I-Lan Longitudinal Aging Study in another region in Taiwan,^16^ might reflect the higher average level of urbanization in New Taipei City (level 2) compared with I-Lan County (level 4)^17^. Elsewhere, frailty is more prevalent among rural older residents in The Republic of Korea than in urban dwellers^18^, and among inhabitants of southern Europe compared to northeners^13^, which may reflect the effect of socioeconomic factors such as education level^13^. The rate of age-demographic shifts accompanying urbanization is an important consideration in disability prevention, although the Taiwan urbanization index already takes into account the prevalence of older adults. Given that frailty arises from accumulated physiological deficits^19^, it is unsurprising that frailty is more prevalent in individuals with multimorbidity than without. Results from the study showed that those vulnerable seniors with multimorbidity, mental disease and residing in less urban areas would experience frailty earlier than the counterparts. Which findings were similar with previous studies from Barnett *et al*.^3^.

When targeting frailty in disability prevention or intervention, identifying subgroups in which the largest effect is likely to result, may be the most pragmatic approach in real-world settings with limited resources. Methods for assessing additive interaction, such as SI, AP and RERI^20^, can help to achieve this objective. Our finding that the combined effect of mental disorders and frailty on functional dependence is more than three-fold higher than these risk factors individually, highlights the importance of addressing mental health in frailty interventions, besides the potential impact of cognitive frailty. In an exploratory factor analysis of 1284 community-dwelling adults, neuropsychological components such as physical health, cognition, or life stress were essential components of broadly-defined frailty^8^. In a population-based study of 715 old adults with 6-year follow up, personal mastery attenuated the impact of frailty on functional decline, consistent with our findings^7^. A recent systemic review showed that training to enhance cognition can diminish frailty^21^. Frailty is a principal target for disability prevention, and our results suggest that early identification of people with dual risks of frailty and mental disorders, as well as instituting comprehensive management of both physical and cognitive frailty, will be crucial to targeting disability prevention and intervention.

In contrast to comorbidity which is a disease-centric concept, multimorbidity implies a patient-centred model of care, which does not prioritize a particular index disease and entails more comprehensive evaluation of individual complexity^22^. Taiwan has universal National Health Insurance that provides prompt and affordable modern health care^23^; nevertheless, we found that multimorbidity and urbanization level affect the prevalence of frailty and have a synergistic effect on disability. This indicates an unmet need for comprehensive public health and medical care focused on integrated functional and disease management in response to population aging with complex care needs^4^.

This study had several noteworthy limitations. First, SI only assesses the additive effect of risk factors and, due to the cross-sectional design, it was not possible to establish the causality of relationships between frailty, mental disorders, multimorbidity, or urbanization. Second, the urbanization index was created more than 10 years ago and might not current urbanicity, due to rapid demographic changes and population aging. Third, although New Taipei City has substantial differences between more and less urban townships, the data derived from citizens of a metropolis might underestimate the synergic effect of urbanization and frailty on disability. Forth, disability was defined as independent living instead of measures of activity of daily living limited to database and many excluded variables due to missing values from living registration might influence generalizability.

Frailty in old people is prevalent and significantly associated with disability, especially among vulnerable groups who have multimorbidity and/or mental disorders, or live in less urbanized areas. Targeting disability prevention requires comprehensive strategies that support clinicians to provide a function-centered model of care that encompasses both physical and mental health, especially in rural areas.

## Methods

### Study design and participants

This study in 2016 retrieved cross-sectional data from the New Taipei City Elderly Health Examination Database, which was established to facilitate earlier detection of physical conditions and promote health in senior citizens. All New Taipei City residents aged ≥65 years were encouraged to have a voluntary examination, paid for by the local government, which included physical examination and biochemistry assays following a standard protocol in medical facilities contracted by the municipal government. New Taipei City Department of Health removed all potentially identifying data to protect privacy and to generate the anonymized secondary Elderly Health Examination Database, which comprised data from 26,026 participants.

When every older participant enrolled, written informed consent is obtained to authorize the New Taipei City Government Institution to process health examination data for the research and policy purpose. New Taipei City Department of Health approved the anonymous use of this dataset for research purposes, and waived the requirement for Institutional Review Board approval stipulated by local government regulations. The study was designed and conducted in accordance with the principles of the Declaration of Helsinki; the cross-section and observational design and reporting format follow Strengthening the Reporting of Observational Studies in Epidemiology guidelines^24^.

### Frailty

Frailty was assessed based the Cardiovascular Health Study (CHS) criteria, which comprise unintentional weight loss, exhaustion, weakness, slowness, and low activity^5^. We defined unintentional loss of weight as more than 5% of total body weight in the previous 12 months. Exhaustion was defined if participants affirmed that two items from the Center for Epidemiologic Studies Depression Scale questionnaire^25^ –“I felt everything I did was an effort” and “I could not get going” – applied for ≥3 days per week. Physical activity, expressed as weekly energy expenditure, was calculated from the International Physical Activity Questionnaire–Short Form score, based on self-reported exercises and leisure time physical activities^26^; energy expenditure below 383 kcal/week in men or 270 kcal/week in women was defined as low physical activity. Weakness was defined as maximum dominant handgrip strength of < 26 kg for men or < 18 kg for women^27,28^. Slowness was defined 6-meter walk speed < 0.8 meter/second, from a static start and with a non-decelerating stop^27^. Participants with three or more of the five CHS frailty components were classed as frail, those with one or two as prefrail, and those with none as being robust.

### Urbanization and population aging

Urbanization level was determined according to criteria published by the Taiwan National Health Research Institute. Based on year 2000 national census data on population density, education level, medical resources, agricultural employment, and population aging, 359 regions throughout Taiwan were classified into seven strata from most urbanized (level 1) to the least (level 7)^17^. To fit the Rothman synergy index (described below), urbanicity was dichotomized into urban (level 1–3) or rural (level ≥4), based on an earlier study^29^; however, none of the 29 municipal districts that constitute New Taipei City were designated as level 6 or 7. The prevalence of people aged ≥65 years residing in more or less urbanized areas and the trend from 2011 to 2016 were used as population aging metrics.

### Other variables

Participants were categorized into four age groups: 65–69,70–74,75–79, and ≥80 years. Multimorbidity was defined as having two or more among specific chronic conditions: hypertension, diabetes, dyslipidaemia, heart disease, kidney disease, stroke, hepatitis B or C, psychiatric disease, rheumatic arthritis, osteoporosis, and history of fractures^3^. Mental disorder was defined as self-reported depression, dementia, or acute psychological stress. Disability was defined as answering no to the question, “do you live independently?”. Smoking and drinking were defined as having partaken of either tobacco or alcohol during the previous 6 months.

### Statistical analysis

Continuous variables were expressed as means plus/minus standard deviation and categorical variables as frequency/proportions. Descriptive characteristics were compared by one-way ANOVA, Student t test, chi-square analysis, or Fisher exact test, as appropriate. Univariate and multivariate logistic regression was used to explore associations between corresponding variables and functional dependence (disability). Rothman *et al*. posit that additive rate or risk models should be used to assess interaction^30^, three such measurements being relative excess risk due to interaction (RERI), the attributable proportion due to interaction (AP), and the synergy index (SI),^20,30^ which is a simple way to assess the size and significance of interactions between candidate binary factors^31^. To assess interaction between factor A and B, subjects with neither are taken as a reference and the respective odds ratios (OR) are estimated: OR_01_ for A, OR_10_ for B, OR_00_ for neither, and OR_11_ for A and B combined; SI is the ratio between OR_11_ and their individual effects.

$$Synergy\,Index=\frac{O{R}_{{\rm{11}}}-1\,}{(O{R}_{{\rm{01}}}-1)+(O{R}_{{\rm{10}}}-1)}$$

SI only examines whether associated factors increase or reduce risk, with no assumptions about causality: SI > 1 indicates positive (synergistic) interaction, SI = 1, indicates no, or exactly additive, interaction, and SI < 1 indicates negative interaction.

RERI indicates the excess portion of the total effect due to interaction, defined as: RERI = 1 + OR_11_ − OR_01_ − OR_10_. RERI = 0 means no interaction or exact additivity, RERI > 0 means positive interaction or synergy, and RERI < 0 denotes negative interaction.

AP is defined as:

$$Attributable\,Porportion=\frac{1+{{\rm{OR}}}_{{\rm{11}}}-{{\rm{OR}}}_{{\rm{01}}}-{{\rm{OR}}}_{{\rm{10}}}}{O{R}_{{\rm{11}}}}$$

As for RERI, AP = 0 means no interaction or exact additivity, AP > 0 means positive interaction or synergy, and AP < 0 denotes negative interaction.

A two-sided p-value of <0.05 or 95% Confidence Interval (CI) that did not span the null hypothesis value were considered statistically significant. All analyses were performed using SAS statistics software, Version 9.4 for Windows (SAS Institute, Cary, NC, USA).

## Data Availability

The data used in this study were sourced from Department of Health, New Taipei City Government, Taiwan. Department of Health, New Taipei City Government does not permit external sharing of any of the data elements. No additional data available.
